# Long COVID-19 and mnemonic effects: an integrative literature review

**DOI:** 10.1590/1806-9282.20231211

**Published:** 2024-04-22

**Authors:** Wóquiton Rodrigo Marques Martins, Tarcísio Viana Cardoso, Ana Lívia Oliveira, Guilherme Silva Fernandes, Ione Fernanda Lemos Fontes, Jaqueline Gonçalves Dantas, Joyce de Souza Miranda, Julio Emanuel Martins, Lorenna Nascimento Antunes, Tarcisio Gomes Leite

**Affiliations:** 1 Centro Universitário Faculdade Guanambi - Guanambi (BA), Brazil.

## INTRODUCTION

The COVID-19 epidemic emerged in Wuhan in December 2019, soon becoming a pandemic with millions of global deaths. Due to the rapid spread of the disease, collapse of health systems, and lack of treatment, radical measures were adopted, such as quarantines, in order to minimize impacts, especially on risk groups^1^.

From this pandemic, the post-COVID syndrome or long COVID-19 emerged, clinically defined by the Delphi method and announced in Geneva^2^. This syndrome generates sequelae in the systems of recovered patients, persisting for more than 3 months after the acute infection, with frequent symptoms such as fatigue and dyspnea. It also causes psychological and neurobiological sequelae linked to the trauma experienced by the patient^3^.

Recent research suggests a relationship between the immune response to the virus and these effects, showing that COVID-19 damages the central nervous system, affecting cognitive functions such as memory. It was noted that 67% of patients affected by long COVID-19 had “brain fog,”^4^ impairing concentration and verbal expression, affecting declarative memory, described as a network of synapses that allows retrieving specific information^5^. Therefore, it is important to research the subject to understand whether, in fact, there is a relationship between the disease and cognitive impairment. Thus, the gap of uncertainty about this cause-consequence will be closed.

## METHODS

This is an integrative literature review, whose guiding theme was the pathophysiological effects of long COVID-19 on memory. This research model is presented as a descriptive study, which consists of the precise detailing of certain facts and phenomena of reality^6^. We opted for an integrative review to obtain a holistic and critical view of the topic^7^. In addition, as only public data were used, there was no need to submit the work to ethical appreciation.

Thus, research was carried out in the bases: BVS, SciELO, MEDLINE, Periódicos CAPES, and Scopus, using a strategy with descriptors: (COVID OR SARS-CoV-2) AND long-term effects AND memory AND (neurology OR psychiatry OR psychology OR cognition OR cognitive functions OR mental health OR brain). This strategy was based on the search for DeCS/MesH descriptors in the Portuguese, English, and Spanish versions. Moreover, the literature review on the mnemonic effects of post-COVID syndrome was enhanced by incorporating the clinical definition established through the Delphi Consensus, along with a thorough exploration of credible scientific sources.

Inclusion criteria for papers were as follows: (a) studies addressing the persistent effects of COVID-19 or SARS-CoV-2; (b) studies published and indexed in peer-reviewed scientific journals; (c) studies that examined memory. Furthermore, as exclusion criteria, the following were applied: (a) observational studies that do not examine memory through recognized methods; (b) studies that were conducted in animals or cells in culture; (c) studies whose sample has diseases that serve as confounding factors for changes in memory; and (d) studies in which socio-environmental aspects (such as social isolation) altered by COVID-19 were related to memory impairment.

The screening of articles was carried out by all authors, independently, using pre-defined criteria. To avoid discrepancies in choices, all screening results were evaluated by the two supervisors, one of whom is a specialist in neurology, regarding study designs, evaluation tools, results, and discussion, in addition to the conclusion, in order to filter the most suitable materials for the research in question.

After analyzing the complete articles, a PRISMA flowchart of the selection process and a table with a summary of the information of the 13 chosen articles were created, using the following parameters: year of publication; authors; title; database; study design; place of study; results; and considerations.

Finally, the data and information in the table were structured in order to guide the analysis and discussion of the results obtained. Such structuring aimed at composing a core of integrated knowledge about the effects of long COVID-19 on memory.

## RESULTS

**Figure 1.**
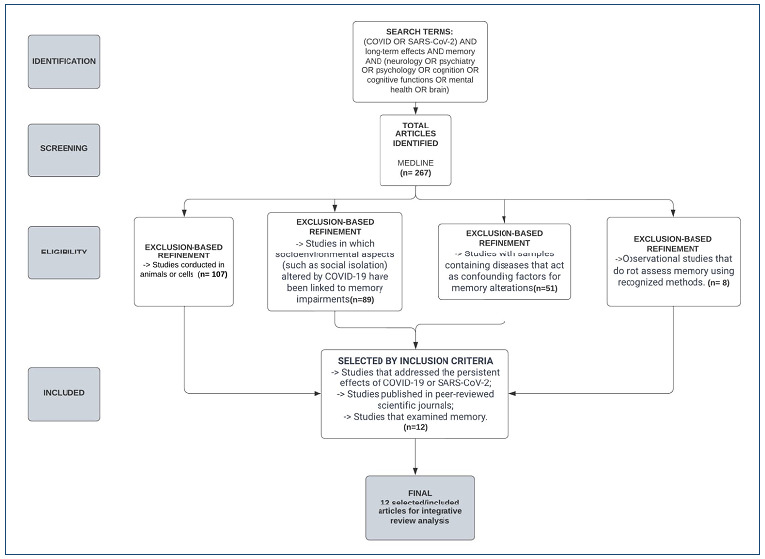
Article selection flowchart. Source: realization by the authors themselves, 2023.

In total, 267 articles were identified in MEDLINE and underwent a screening stage, as illustrated in Figure 1. From this group, the following were excluded: 107 studies involving animals or cell cultures; 89 articles focused mainly on the socio-environmental impacts of the COVID-19 pandemic on memory; 51 articles that presented confounding factors in the analysis of the relationship between COVID-19 and memory, such as neurodegenerative diseases; and seven observational studies that did not investigate memory by recognized clinical or laboratory methods. Finally, 13 articles that were published between 2021 and 2023 met the criteria and are summarized in Table 1.

**Table 1.** Summary of review articles.

Year	Authors	Title	Kind of study	Place of study	Results	Considerations
2021	Hellmuth J. et al.^9^	Persistent COVID-19-associated neurocognitive symptoms in non-hospitalized patients.	Case series.	Ambulatory.	Of the first 100 participants evaluated, 20 had cognitive problems at one or more study visits, 12 had cognitive symptoms at the baseline visit, and 8 had no baseline symptoms but noticed during follow-up.	Neurocognitive symptoms associated with COVID-19 have been observed in young and middle-aged adults who have not undergone hospitalization.
*2021*	Graham EL. et al.^8^	Persistent neurologic symptoms and cognitive dysfunction in non-hospitalized Covid-19 “long haulers”.	Prospective cohort.	Neuro-Covid-19 Clinic of Northwestern Memorial Hospital (EUA).	Non-hospitalized SARS-CoV-2 infected had working memory deficit at follow-up, relative to the US median (T-score 43 vs 50, p = 0.007) in the NIH Toolbox test.	About 80% had neurological impairment, 50% with changes in short-term memory and attention.
2022	Bungenberg J. et al.^10^	Long COVID-19: Objectifying most self-reported neurological symptoms	Transversal.	RWTH Aachen University Hospital, Germany.	Patients reported difficulties with attention (56%), memory (38%), and word search (18%). Hospitalized had worse performance in MoCA, logical reasoning, and verbal memory.	The authors point to mild deficits in attention, processing speed, and memory in cognitive performance. Some had severe impairment in attention and executive functions.
2022	Premraj L. et al.^11^	Mid and long-term neurological and neuropsychiatric manifestations of post-COVID-19 syndrome: A meta-analysis.	Systematic review.	-	The overall prevalence of memory problems was 28% (22-35%) in a total sample of 10,530 properly screened patients.	Memory impairment stood out even 3 months after the onset of the acute illness.
2022	Crivelli L. et al.^12^	Changes in cognitive functioning after COVID-19 A systematic review and meta-analysis	Systematic review.	-	In the study, memory was one of the most affected domains, in which delay and impairment of immediate verbal memory were noted. In the longer follow-up study, deficits in memory coding were found.	The meta-analysis provided preliminary evidence suggesting that individuals may exhibit reduced cognitive performance in some domains after recovery from COVID-19.
2022	Nakamura A. et al.^13^	Long-Term Sequelae in Young Convalescent COVID-19 Patients.	Prospective cohort.	Duke University Memory Disorders Clinic.	The three patients scored above 26 on the MoCA. All were diagnosed with subjective cognitive impairment. Neuropsychological tests a month later showed improvement, except in verbal memory.	The authors conclude that young patients with cognitive sequelae of SARS-CoV-2 infection symptoms tend to improve with time.
2022	Keijsers K. et al.^19^	Memory impairment and concentration problems in COVID-19 survivors 8 weeks after non-ICU hospitalization: A retrospective cohort study.	Retrospective cohort.	Jeroen Bosch Hospital.	Among symptoms 8 weeks after hospitalization, complaints of memory impairment were 33%.	Memory issues are common post-COVID, so there should be extensive care for such affected patients.
*2022*	Zawilska JB, Kuczyńska K.^16^	Psychiatric and neurological complications of long COVID.	Integrative review.	-	Memory impairment was observed in different age groups. In adults, deficits in short-term memory and general memory loss have been observed.	Neurological symptoms seem to be characteristic of long-term COVID, with cognitive deficits being the most recurrent.
2022	Soh HS, Cho B.^15^	Long COVID-19 and Health-Related Quality of Life of Mild Cases in Korea: 3-Months Follow-up of a Single Community Treatment Center.	Prospective cohort.	Seongnam Community Treatment Center, in South Korea.	Factors linked to persistent symptoms and reduced quality of life are: female gender, metabolic disease, and anxiety in the acute phase of COVID-19.	At baseline, 89.1% of patients had one or more symptoms. The most common persistent symptoms were memory impairment, fatigue, and loss of quality of life.
2022	Perrottelli A. et al.^14^	Cognitive Impairment after Post-Acute COVID-19 Infection: A Systematic Review of the Literature.	Systematic review.	-	Memory is among the most affected cognitive domains.	A relationship was noted between SARS-CoV-2 infection and the emergence of memory deficits.
2023	Cavaco S. et al.^18^	Predictors of Cognitive Dysfunction One-Year Post COVID-19.	Retrospective cohort.	Porto University Hospital Center.	After 1 year, more than 50% of patients had abnormal performance on at least one cognitive test and more than one-third had significant cognitive complaints.	Part of patients with long-term COVID have persistent cognitive changes
2023	Taruffi L. et al.^17^	Neurological Manifestations of Long COVID: A Single-Center One-Year Experience.	Retrospective observational	University of Bologna, Italy.	Out of 130 patients, 46 had cognitive disorders. Among the 30 assessed, 37% had changes in memory; 86.4% had acute neurological symptoms.	Of the cognitive abilities, memory was the most impaired, with a prevalence of 37% of patients.
2023	Gamberini J. et al.^20^	Previously independent patients with mild-symptomatic COVID-19 are at high risk of developing cognitive impairment but not depression or anxiety.	Prospective observational.	Unit of the “Mons. L. Novarese” Rehabilitation Center (Moncrivello, Italy).	In the MoCA test, 81.1% of the patients had impaired memory, specifically in 76.7% of the youngest and 83.3% of the elderly (>65 years).	In the MoCA test, the elderly had a higher prevalence of mild cognitive impairment in memory.

Source: realization by the authors themselves, 2023.

## DISCUSSION

The National Institute for Health and Care Excellence (NICE) guidelines define post-COVID-19 syndrome as “signs and symptoms that develop during or after an infection consistent with COVID-19, that persist for more than 12 weeks and are not explained by an alternative diagnosis.”

Memory, which is the central focus of this study, is defined as a facet of cognition, similar to learning, attention, language, and executive function. Thus, a cognitive deficit indicates a reduction in a previously normal domain, negatively affecting the individual’s daily life.

In a prospective cohort, developed in 2020, 100 patients were analyzed, without previous hospitalization for pneumonia or hypoxemia and with clinical manifestations of COVID-19 compatible with the guidelines of the Infectious Diseases Society of America (IDSA) for at least 6 weeks^8^. Upon confirmation of diagnosis using reverse transcription polymerase chain reaction (RT-PCR), 50 patients tested positive for SARS-CoV-2, and the other 50 tested negative for SARS-CoV-2. A complete neurological examination was performed on the 52 patients who attended the neurology clinic and a limited neurological examination on the 48 patients who were followed up virtually. Overall, 53% had an abnormal examination, with the most frequent neurological signs of short-term memory deficit (32%) and attention deficit (27%).

On another occasion, a case series was conducted, presenting two reports of female patients recovering from COVID-19, without hospitalization, with persistent neurocognitive symptoms^9^. The first patient, who was evaluated in a memory clinic, mentioned that cognitive symptoms appeared in the first week, including difficulty concentrating, and memory problems improved by cues and information processing. Despite showing deficits in working memory, she scored 30/30 on the Montreal Cognitive Assessment (MoCA). The second patient, who was assessed by telemedicine 37 days after the onset of COVID-19 symptoms, scored perfect (30/30) on the Mini-Mental State Exam (MMSE). However, she reported difficulty expressing herself and reduced organization, leading to missed deadlines. In conclusion, the need for further research to determine the prevalence of neurocognitive symptoms associated with COVID-19 was highlighted.

A cross-sectional study was carried out with 50 patients after infection with SARS-CoV-2 having persistent symptoms for at least 4 weeks^10^. These patients were divided into hospitalized (n=21) and non-hospitalized (n=29). A neurologist conducted complete neurological examinations and structured interviews. Verbal episodic memory was assessed using the verbal auditory memory test (VLMT) or Word List from the CERAD-Plus Battery. Visual memory was analyzed with the Rey-Osterrieth complex figure test (ROCFT) or CERAD-Plus figure subtest. Patients mentioned memory complaints (38%) and word search problems (18%). Neuropsychological performance, in general, remained within the reference standards. Although hospitalized patients had lower verbal performance, cognitive performance was comparable between groups and, for the most part, without impairment. However, the study has limitations due to the small sample size and the absence of a control group.

In a future meta-analysis, 18 studies were included in the systematic review, which included 10,530 patients for the final analysis^11^. We determined the prevalence of neurological and neuropsychiatric symptoms reported from 3 months after acute onset of COVID-19 in adults. A mean prevalence of memory problems was obtained around 28% of the patients, with a variation between 22 and 35%. The authors came to the conclusion that cognitive dysfunction (brain fog, memory problems, and attention disorder) was the main neurological feature present in patients with long-term COVID-19.

A pooled analysis of 27 studies involving 2,049 participants and an additional study with 16 moderate to severe SARS-CoV-2-positive patients revealed that one of the most affected cognitive domains is immediate verbal memory (38% moderate and 11.2% severe)^12^. The larger study, which followed patients over 7 months and included 749 participants, also found memory coding deficits in 24% (n=178) and memory recall deficits in 23% (n=170). However, the authors of the systematic review highlight the scarcity of articles to conclude how the severity of COVID-19 or the types of symptoms directly influence cognition and, consequently, memory.

During the conduct of a case series, which included three reports of female patients under the age of 60 years, who sought the Duke University Memory Disorders Clinic due to complaints of cognitive dysfunction after recovering from SARS-CoV-2 infection^13^, the Gold Standard MoCA test or a follow-up neuropsychological test was administered to all three patients. The first patient scored 28/30 on her initial assessment and 4 months later achieved a score of 30/30. At the baseline visit, the second patient achieved a MoCA score of 29/30, and after 1 month, during follow-up, she demonstrated normal general cognitive function. The third patient received a MoCA score of 26/30 during the initial assessment and underwent neuropsychological tests a month later, which revealed difficulties in verbal memory. In summary, neuropsychological findings suggest that patients who experience cognitive sequelae after SARS-CoV-2 infection tend to experience improvement in their symptoms over time.

Some studies broadly address cognitive impairments resulting from COVID-19. Focusing on memory for this integrative review, in studies such as the one mentioned above, an attempt was made to assess the impacts on memory in comparison with other cognitive areas. In this context, memory proved to be one of the main affected aspects, which was individually considered in 37 studies for the preparation of a systematic review^14^, of which 30 indicated some degree of impairment. The conclusion highlighted by the authors is that all subdivisions of memory showed some level of impairment.

As can be observed, there are several implications of the post-COVID syndrome on the quality of life of patients, caused by neurological depression and neuropsychiatric sequelae.

Another cut proposed in the analyzed studies is that of age groups, which describe the occurrence of systemic dysfunctions in different age groups^15^. When considering children as less affected by COVID-19 infection, therefore, they are the least affected by long-term COVID-19. In young and elderly adults, the picture is different. The study points out, within memory impairment, that the highest percentage of adults is affected by “short-term” memory loss. In this population, the problems are accumulated, and when considering the multifactorial nature of the post-COVID syndrome, the consequences are greater. The author mentions that cognitive impairment even hinders the return to work.

We analyzed 103 patients who attended an outpatient clinic for their first consultation, on average 243 days after the initial diagnosis of COVID-19^16^. Among the evaluated patients, 30 were submitted to the application of neurological scales, such as the MoCA. The results of the analysis indicated that the most impaired cognitive function was memory, affecting approximately 37% of patients. These findings may be related to later findings, which also observed neurological impairments in COVID-19-infected patients, however, in a period of at least 1 year after infection^17^. In this context, it is possible to identify similarities in the results obtained, and the main discrepancy is the average time interval for the onset of symptoms.

The studies in this literature review help clarify the connection between coronavirus infection and later-stage memory dysfunction. However, due to the recency of events, it is likely that the evidence confirming the causal link between post-COVID syndrome and memory problems will strengthen over time.

By establishing inclusion and exclusion criteria that prioritized objective and quantitative research, the researchers limited the scope of the study, neglecting investigations that integrated social determinants into the analysis of memory. It is important to highlight that, even given the scarcity of articles on the topic, post-COVID syndrome has emerged as a triggering factor for complex complications in the memory of individuals affected by this specific condition. Therefore, we suggest a reflection on the need for more comprehensive and holistic approaches to fully understand the impacts of the syndrome on the mnemonic function, considering both objective aspects and underlying social elements.

## CONCLUSION

The study aimed to research the connection between SARS-CoV-2 infection and cognitive dysfunctions, using MEDLINE sources. However, reaching a broad conclusion about the pathological impact on memory through the selected articles is complex, due to different participant variables, such as age and severity of cases. There was also variation in location, with frequent mentions of the American, European, and Asian regions, and a slight majority of female participants, with emphasis on adults and the elderly.

Despite the differences, there are some similarities between the articles: mild or asymptomatic cases of the disease can cause cognitive problems, affecting memory and concentration, affecting quality of life^18^, which highlighted the negative influence of neuropsychiatric symptoms on quality of life assessed by the EuroQol-5 dimensions-5 levels questionnaire (EQ-5D-5L). Most patients demonstrated improvement in symptoms over time. Children and adolescents represent a minority in cases of COVID-19 and long-term COVID-19.

In addition, due to the novelty of the topic, the post-COVID consequences are still poorly understood. Most studies^9^ ^,^ ^12^ ^,^ ^13^ ^,^ ^14^ ^,^ ^15^ ^,^ ^16^ ^,^ ^17^ mention possible pathological mechanisms contributing to cognitive symptoms, including direct damage by virus invasion in the central nervous system^9^ ^,^ ^12^ ^,^ ^14^, decreased activity of angiotensin-converting enzyme 2 (ACE-2) - linked to memory changes in the hippocampus^12^ ^,^ ^14^ - and hyper-inflammatory state, increasing levels of inflammatory markers such as cytokines, TNF-a and G-CSF, in addition to high levels of antinuclear antibodies.

Cases showed evidence of changes in the cortex, ranging from atrophy in the orbitofrontal cortex and occipital regions^13^ to white matter abnormalities, impairing the frontal and parietal lobes^14^. Conditions such as decreased lung function, delirium, sepsis, hypoxia, vascular dysfunctions, ongoing endothelial activation, residual immune activation, and residual lesions accumulated during acute illness can all worsen cognitive performance.

Regarding the drug approach, the studies were not carried out in-depth due to the complexity of the possible mechanisms in this clinical condition, which highlights the need for more research.
